# Retraction: Overexpression of TrpC5 promotes tumor metastasis via the HIF-1α/Twist signaling pathway in colon cancer

**DOI:** 10.1042/CS20171069_RET

**Published:** 2025-06-02

This article “Overexpression of TrpC5 promotes tumor metastasis via the HIF-1α–Twist signaling pathway in colon cancer” DOI: 10.1042/CS20171069 is being retracted from *Clinical Science* at the request of the authors and the Editorial Board. This follows receipt of a notification from a reader, alerting the Editorial Board to:

- Figure 2B β-actin SW116 and SW620 protein bands, which also appear as Figure 5A HCT116 ZEB1
- Figure 2D SW620-sh-TrpC5 Migration panel, a part of which also appears in Figure 2D HCT116-LV-TrpC5 Migration panel with rotation
- Figure 5A SW620 Snail, which also appears as Figure 5B GAPDH SW620-siHIF-1α and SW620-siTWIST protein bands
- Figure 5A HCT116 Snail, which also appears as Figure 3D GAPDH WT + NC and WT + siNotch3 protein bands from a previous publication with overlapping authors: Gu et al, 2016 (DOI: 10.1007 /s13277-016-5412-4)
- Figure 5C siHIF1α panel, a part of which also appears as Supplementary Figure S3B HCT116 Control Invasion panel

The authors were contacted regarding the concerns raised and responded that the duplications were due to mistakes in exporting and selecting the images. The authors provided replacement data for the relevant images in Figures 2B, 2D, 5A and 5C. The authors additionally provided replacement data for Figure 4A DAPI/TrpC5 images, as it was noted that Figure 2C SW620-shTrpC5 appears as Figure 4A HTC116 LV-vector, and Figure 2C HCT116 LV-Vector appears as SW620 TrpC5-shRNA. The Editorial Office further noted that Figure 5C SW620 Scramble partially overlaps with Figure S3B HCT116 Invasion hFGF image.

Given the extent of the issues raised, the authors wish to retract the article and state that it is their responsibility to protect scientific rigorism and accuracy of the manuscript. The Editor-in-Chief and Editorial Board agree with the retraction.

